# PLGA nanoparticles as an efficient carrier in *Toxoplasma* GAP45: a more effective vaccine against acute toxoplasmosis than traditional ones

**DOI:** 10.3389/fimmu.2025.1600399

**Published:** 2025-06-23

**Authors:** Pan Zhou, YanLi Yu, WeiYu Qi, XiaoJuan Wang, YouLi Yu, JianDong Wang, Li Zhang, ZhengQing Yu, TingLi Liu

**Affiliations:** ^1^ School of Animal Science and Technology, Ningxia University, Yinchuan, China; ^2^ School of Economics and Management, Ningxia University, Yinchuan, China; ^3^ Institute of Animal Science, Ningxia Academy of Agricultural and Forestry Science, Yinchuan, China; ^4^ Department of Medical Laboratory, Fenyang College of Shanxi Medical University, Fenyang, China

**Keywords:** *Toxoplasma gondii*, glidesome-associated protein 45, PLGA nanoparticles, immunoprotection, mice

## Abstract

**Introduction:**

*Toxoplasma gondii* (*T. gondii*), as a strict intracellular parasite, can infect nearly all mammals, including humans, posing significant threats to public health. Toxoplasmosis in animals also leads to substantial economic losses in animal husbandry. Currently, no effective treatments are available for toxoplasmosis, creating an urgent need for safe and efficient therapeutics.

**Methods:**

In this study, we constructed a subunit vaccine using *T. gondii* glidesome-associated protein 45 (TgGAP45). To enhance immunogenicity, poly (lactic-co-glycolic acid) (PLGA) nanoparticles were employed as delivery carriers to prepare TgGAP45-PLGA nanospheres. For comparison, two oil adjuvants, Montanide™ ISA 660 VG and Montanide™ ISA 206 VG, were used to formulate TgGAP45-206VG and TgGAP45-660VG emulsions. Following safety evaluation, protective immunity was assessed in animals. Antibody levels, cytokine profiles, dendritic cell (DC) maturation and differentiation, and T lymphocyte proliferation and differentiation were analyzed.

**Results:**

The results demonstrated that TgGAP45-PLGA nanospheres induced a mixed Th1/Th2 immune response against *T. gondii*. Furthermore, parasite burden analysis in spleen and heart tissues revealed that TgGAP45-PLGA nanospheres provided the strongest immunoprotection among the tested vaccines.

**Discussion:**

These findings indicate that TgGAP45 delivered via PLGA nanospheres is a promising candidate for preventing acute toxoplasmosis. Further studies and applications are warranted to explore its full therapeutic potential.

## 1 Introduction

The intracellular protozoan *Toxoplasma gondii* was firstly reported over 100 years ago in rabbits (1) and can infect a variety of mammals including humans (2, 3). *T. gondii* infections in newborns can lead to severe congenital defects, even miscarriage and premature birth (4, 5), while the toxoplasmosis that occurred in immunocompromised patients can result in severe consequences, even death (6, 7). Currently, one-third of the world’s population were detected with *T. gondii*-positive antibodies in sera (8), and severe economic losses in livestock cannot be neglected (2). Currently, there is no effective therapeutic method available to treat toxoplasmosis (9), and the most frequently used drugs in clinical patients are pyrimethamine (PYR) and sulfadiazine (SDZ); such strategies are of no use against the bradyzoites of *T. gondii* and have serious side effects (10, 11). To prevent sheep abortion, a vaccine named Toxovax^®^ (Intervet Inc., Angers, France) is licensed by New Zealand (12), but the risk of virulence recovery cannot be ignored (13). In addition, the application of Toxovax^®^ in foodborne animals has been questioned due to the formation of tissue cysts in immunized animals. Therefore, to protect humans and cut economic losses, it is imperative to develop an effective and efficient vaccine against *T. gondii*.

Evidence suggests that the development of effective vaccines could be a critical strategy for controlling *T. gondii* infections (14). With the advancement in the field of vaccinology, various types of vaccines, including DNA vaccines, live attenuated vaccines, and subunit vaccines, have emerged (15), and plenty of vaccines have been proven effective in toxoplasmosis (16, 17). However, all these verified vaccines cannot provide complete immune protection against toxoplasmosis in either humans or animals (18). The core problem of research and development in new vaccines involves the choice of vaccine antigens (19).

Among various *T. gondii*-derived immunogens, surface antigens (SAGs) like SAG1 have been widely studied for their roles in preventing toxoplasmosis (20), while rhoptry proteins (ROPs) and dense granule proteins (GRAs) are known to participate in parasite invasion and intracellular survival (21, 22). As a key protein in peripheral membrane protein, glidesome-associated protein 45 (TgGAP45) can employ the motor complex consisting of myosin A and two related myosin light chains, and is associated with *T. gondii* motility, invasion, and escape from infected cells (23). In addition, TgGAP45, through N-terminal acylations, is anchored to the membrane of *T. gondii* (23), with the C-terminal part connected to TgGAP40 and/or TgGAP50 proteins (24, 25), and such structures are responsible for the gliding motility in *T. gondii* (26). This unique functional involvement makes TgGAP45 distinct from other well-characterized antigens like SAG1 or ROP18, potentially offering broader protective immunity by targeting the fundamental motility machinery. All these studies suggested that TgGAP45 plays an important role in parasite motility and could be a potential antigen for *T. gondii* vaccines.

Currently, DNA vaccines and subunit vaccines are reported to be highly efficient in preventing *T. gondii* infections (27). Limitations also emerged in DNA vaccines such as the risk of gene integration in host genomic DNA (28, 29), and such limitation can be solved by subunit vaccines; it uses a small fraction of parasitic component to induce immunity in vaccines (29). However, the proteins carried by subunit vaccines can be easily degraded *in vivo* and lose their antigenicity. To fix this, numerous kinds of adjuvants were reported, such as liposomes, lipid nanoparticles, and polymeric micelles (30, 31). When evaluating nanocarriers for subunit vaccines, liposomes represent a classical choice with demonstrated high antigen loading capacities (30, 32). However, their phospholipid-based architecture introduces susceptibility to oxidative degradation and colloidal instability during storage. Although these adjuvants could enhance immunoprotection in the host, in almost all cases, subunit vaccines co-immunized with adjuvants cannot provide full protection against *T. gondii* (33). The emergence of nanometer technology brings new opportunities to *T. gondii* subunit vaccines (34). In contrast to these lipid-based systems, poly(lactic-co-glycolic acid) (PLGA) nanomaterials and nanoparticles provide distinct advantages through their Food and Drug Administration/European Medicines Agency (FDA/EMA)-approved status and predictable biodegradation kinetics (35). The polymer’s erosion-controlled release mechanism enables sustained antigen exposure critical for immune memory development, while its glass transition temperature ensures superior storage stability compared to lipid systems (36, 37). In immunizations, longer extension of antigens could generate more effective immune responses, and such nanomaterials could also avoid the potential risk of tolerance, and substitute several enhancing injections typically required to elicit protective immune responses (38). These characteristics, combined with proven safety profiles in clinical application, deem PLGA to be particularly suitable for toxoplasmosis vaccine development where both immunogenicity and long-term stability are paramount (35, 39).

In the current research, we attempted to entrap the recombinant TgGAP45 proteins (rTgGAP45) in PLGA nanomaterials to construct a novel nanoparticle, named TgGAP45-PLGA nanoparticles. In addition, two emulsions entrapped with rTgGAP45 were also prepared for comparison, and these two emulsions were synthesized with two commercial oil-based adjuvants, Montanide™ ISA 660 VG and Montanide™ ISA 206 VG. Based on the reports, two products that we used can result in a strong and long-lasting immune response in immunized animals (40, 41) and can induce short-term and long-term immune responses. After vaccine formulations, we injected the preparations into animals via two doses, and the *T. gondii* loads were analyzed in spleen and heart tissue to demonstrate the immunoprotection of three prepared vaccines. The findings of our research stressed the value of nanoparticles loaded with TgGAP45 proteins in enhancing potential immunity against acute toxoplasmosis.

## 2 Materials and methods

### 2.1 Animals and parasites

As a relevant model used in this study, female ICR mice weighing 18–22 g (7 weeks old) were purchased from Vital River Laboratory Animal Technology Co., Ltd. (Beijing, China), and were cultured at a specific pathogen-free (SPF) environment. Purchased from the same company, female Wistar rats weighing 180–220 g (7–8 weeks old) were also cultured in the same SPF environment. The experimental design, animal management, injection, and operations strictly followed the Ethics Procedures and Guidelines of the People’s Republic of China and were supervised by the Animal Ethics Committee, Ningxia University, Yinchuan, China.

The purified *T. gondii* RH strains were obtained from Dr. Xiangrui Li at the Nanjing Agricultural University, and retained in liquid nitrogen at the Preventive Veterinary Laboratory, College of Veterinary Science, Ningxia University, Yinchuan, China. The parasites were propagated in mice as previously described (42).

### 2.2 Acquisition of recombinant TgGAP45 protein

The total RNA of 10^7^ *T. gondii* tachyzoites was purified by Trizol reagent (TAKARA Biotech, Dalian, China), and the cDNA was synthesized by using the reverse transcription kit (TAKARA Biotech, Dalian, China). PCR amplification (Applied Biosystems, Waltham, MA, USA) was then conducted to obtain the conserved domain sequences (CDS) of TgGAP45 (GenBank: TGME49_223940), using the following primers: forward, 5'-CCATGGCTGATATCGGATCCATGGGAAACGCGTGCAAGA-3', and reverse, 5'-TGTCGACGGAGCTCGAATTCTCAGTTCAACAAGGGTGCATCC-3'. Reverse PCR was also conducted with the blank pET-32a plasmid as the template by using the following primers: forward, 5'-GAATTCGAGCTCCGTCGACAAGC-3', and reverse, 5'-GGATCCGATATCAGCCATGG-3'. Phanta Max Master Mix (BioGlod Science, Zhejiang, China) was used for the PCR amplification mentioned above with the recommended protocol. Then, the recombinant plasmid, named pET-32a-TgGAP45, was constructed by using the homologous recombination system (Vazyme Biotech, Nanjing, China), and was transferred into *Escherichia coli* BL21 (DE3) cells (Tsingke Biotech, Xian, China). The *E. coli* BL21 (DE3) cells carrying pET-32a-TgGAP45 plasmids were propagated in Luria Bertani (LB) medium containing 100 μg/mL ampicillin. The plasmids were extracted by a commercial reagent kit (Omega Bio-Tek, Norcross, GA, USA), verified by double restriction enzyme digestion (TAKARA, Dalian, China), and sequenced by the ABI PRISM™ 3730 XL DNA Analyzer (Applied Biosystems, Waltham, MA, USA). To collect purified recombinant TgGAP45 proteins, BL21 (DE3) cells were cultured in LB medium containing 100 μg/mL ampicillin, and 1.0 mM isopropyl β-D-thiogalactoside (IPTG, Aladdin, Shanghai, China) was added until the OD_600_ (optical density at 600 nm) reached approximately 0.5. The chemical competent cells were collected, crushed by tip sonication (Scientz Biotechnology, Ningbo, China), and purified by a chelating column (Cytiva, Marlborough, MA, USA). Before subsequent analysis, the endotoxin in purified rTgGAP45 was eradicated by the Endotoxin Removal Kit (GeneScript, Piscataway, NJ, USA), and the endotoxin level of rTgGAP45 after eradication was detected by a commercial kit (GeneScript, Piscataway, NJ, USA). To analyze the purification and concentration of purified rTgGAP45, 12% (w/v) sodium dodecyl sulfate–polyacrylamide gel electrophoresis (SDS-PAGE) and the BCA assay were performed.

### 2.3 Immunoblot detection

To collect the sera against rTgGAP45, purified rTgGAP45 was emulsified with an equal volume of Freund’s complete adjuvant (Sigma-Aldrich, St. Louis, USA), and each Wistar rat received a subcutaneous injection of emulsions containing 200 µg of purified rTgGAP45 in the back skin with multiple points in the first week. The emulsion mixture of purified rTgGAP45 and Freund’s incomplete adjuvant (Sigma-Aldrich, Saint Louis, USA) was vaccinated four times into rats through the same method mentioned above at 14-day intervals. Whole blood was harvested 1 week later after the last vaccination from the orbit, and the sera were separated and stored at −20°C. Blank sera were also collected from animals immunized with PBS using the same vaccination strategy. Furthermore, the soluble tachyzoite antigen (STAg) of the parasites was prepared as described previously (43). Briefly, 10^8^ *T. gondii* tachyzoites were collected and resuspended in RIPA lysis (Biosharp, Life Science, Hefei, China), then tip sonication was conducted. To obtain the sera against *T. gondii*, rats were injected with emulsions containing STAg and Freund’s adjuvant using the same immunization strategy. All sera were stored at −20°C until use.

Purified rTgGAP45 and STAg were first separated in 12% SDS-PAGE gel and were subsequently transferred to polyvinylidene fluoride (PVDF) membranes (Millipore Ltd., Tullagreen, Carrigtwohill, Co. Cork, IRL) via the Mini Trans-Blot Cell (Bio-Rad, Hercules, CA, USA). Membranes were then blocked in TBST [tris-buffered saline containing 0.5% (v/v) Tween 20] containing 5% (w/v) skimmed milk powder (Sangon Biotech, Shanghai, China) at 37°C for 2 h, and were separately incubated in TBST containing rats’ sera against STAg and in the TBST containing the sera against rTgGAP45 at 1:100 dilutions overnight at 4°C on a rotary shaker. Rinsed three times in TBST, membranes were incubated with horseradish peroxidase (HRP)-conjugated anti-rat IgG (1:5,000 dilutions, ABclonal Technology, Wuhan, China) for 2 h at room temperature. Finally, the membranes were visualized by the Electro-Chemi Luminescence (ECL) system (Tanon, Shanghai, China). The sera harvested from animals immunized with PBS were also conducted as a control.

### 2.4 Synthesis of nanoparticles and vaccines

Based on the double emulsion solvent evaporation technique (44), the TgGAP45PLGA nanoparticles (w/o/w nanoparticles) were synthesized with minor modifications. In short, to form PLGA solution, 0.50 g of PLGA (MW: 40,000–75,000 Da, LA/GA: 65/35, Macklin Biochemical Technology, Shanghai, China) was dissolved in 10.0 mL of dichloromethane (DCM, Macklin Biochemical Technology, Shanghai, China) at room temperature. Then, 20 mL of 5% (w/v) polyvinyl alcohol (PVA, MW: 31,000–75,000 Da, Macklin Biochemical Technology, Shanghai, China) was added dropwise into the PLGA solution under frequent stirring. The mixture was then kept in an ice bath, and tip sonication was carried out in continuous mode (duration, 2 s; interval time, 3 s) under an output power of 40 W, until the mixture turned milky white. After that, 40 mg of rTgGAP45 without endotoxin was dropwise added under frequent stirring at room temperature, and tip sonication was again conducted under the same criteria. To form w/o/w emulsions, 20 mL of 5% PVA was subsequently dropwise added, and tip sonication was performed again. Before centrifugation at 35,000 rpm for 20 min at 4°C, the obtained emulsions were passed through the 0.22-μm filter membrane (Millipore, Billerica, MA, USA) to remove unexpected impurities. The precipitates were collected and resuspended in deionized water, while the supernatants were also harvested. The resuspended solution was stored at −20°C overnight and was completely freeze-dried (Labconco, Kansas City, MO, USA) to remove DCM. The freeze-dried powder, also known as TgGAP45-PLGA nanoparticles, was stored at −20°C and diluted by 1 × PBS before use. To characterize the sustained-release properties of the nanoparticles, an *in vitro* release study was conducted under sink conditions. The experiment utilized PBS (pH 7.4) as a release medium with a volume exceeding 10-fold the saturation solubility of rTgGAP45, ensuring the protein concentration remained below 10% of its saturation limit throughout the assay. To accurately mimic physiological conditions, the release medium was maintained without replacement during the entire study period.

To obtain the TgGAP45-206VG emulsions (w/o/w emulsions), Montanide™ ISA-206VG (Seppic, Paris, France) was pre-incubated in a 50°C water bath before use, and the rTgGAP45 was dissolved in PBS (pH 7.4) to a final concentration of 1.0 mg/mL. According to the guidelines, the protein solution was gently added into Montanide™ ISA-206VG in equal volumes at a bath temperature of 31°C.

Afterward, the emulsions were stirred constantly until thickened at a temperature of 31°C. The formulated TgGAP45-206VG emulsions were shortly stored at 4°C until use (within 2 h).

To obtain the TgGAP45-660VG emulsions (w/o emulsions), the rTgGAP45 was dissolved in PBS (pH 7.4) to a final concentration of 1.0 mg/mL. Based on the instructions, the protein solution was directly added into Montanide™ ISA-660VG in a 2:3 (w/w) ratio at room temperature. Then, the emulsions were stirred constantly until thickened, and the synthesized emulsions, also known as TgGAP45-660VG emulsions, were also shortly stored at 4°C until use (within 2 h).

### 2.5 Nanoparticle characterization

To analyze the surface morphology of TgGAP45-PLGA nanoparticles, the synthesized nanoparticles were imaged by a scanning electron microscope (SEM, SU8010, Hitachi, Tokyo, Japan) at Nanjing Agriculture University (Nanjing, China). TgGAP45-PLGA nanoparticles in the SEM images were randomly measured, and the average diameter of synthesized nanoparticles was calculated by ImageJ software (version 1.8.0, NIH, Bethesda, MD, USA). Using Zetasizer nanoseries nano-ZS (Malvern Panalytical Ltd., Malvern, UK), the polydispersity index (PDI) of synthesized nanoparticles was examined using the dynamic light scattering technique in Saisijuxin Biotechnology (Yinchuan, China).

To investigate the encapsulation efficiency (EE) of prepared nanoparticles, the synthesized TgGAP45-PLGA nanoparticles were separated from the supernatant via high-speed centrifugation at 40,000 rpm for 20 min at 4°C (as described in Section 2.4). The total volume of the supernatant was evaluated by a measuring cylinder, and EE was calculated based on Equations 1 and 2.


(1) $$\begin{array}{c} \text{Free}\;\text{protein}\;\left(\text{mg}\right)\;\\ =\;\text{Free}\;\text{protein}\;\text{concentration×Supernatant}\;\text{volume} \end{array}$$

(2) $$\text{EE}\;\left(\%\right)=\frac{\text{Weight}\;\text{of}\;\text{nanoparticles-Free protein}}{\text{Weight}\;\text{of}\;\text{nanoparticles}}\mathrm{x100\%}$$

To investigate the sustained release behavior of TgGAP45-PLGA nanoparticles, the cumulative release assay was also performed *in vitro* according to the previously reported method with minor modification (45). Synthesized nanoparticles were dissolved in 1 × PBS (pH 7.4) and kept on a shaker under 37°C in the centrifuge tubes, then 20 μL of supernatant as the sample was collected at an interval of 12 h after centrifugation at 15,000 rpm for 20 min. Nanoparticle solution was resuspended and the total volume was recorded; samples were kept at −20°C until use. After the last record, the total concentration of free proteins in the samples was demonstrated by BCA assay, and the *in vitro* cumulative release (CR) profile was constructed by using Equation 3. A least-square 233 fitting method was conducted to construct the fit line.


(3) $$\text{CR(\%)=}\frac{\text{Total}\;\text{volume}\;\times \;\text{Protein}\;\text{concentration}}{\text{Total}\;\text{loaded}\;\text{proteins}}x100\%$$

To demonstrate the toxicity of TgGAP45-PLGA nanoparticles, 25 ICR mice were randomly divided into four groups: Blank (administered with equal volume of 1 × PBS), Control (administered with pET-32a vector protein), TgGAP45 (administered with rTgGAP45), and TgGAP45-PLGA (administered with TgGAP45-PLGA nanoparticles). Each animal received a subcutaneous injection with a dose containing 300 μg of antigen, which was three times higher than usual. A booster vaccination was also conducted 7 days later using the same strategy, and the sera were harvested from the eye sockets of animals. Based on the indophenol blue method and the creatine oxidase 234 method, the concentrations of blood urea nitrogen (BUN) and creatinine (Cr) were analyzed by the 250 commercial kits (Solarbio, Beijing, China). During the trials, the animals’ physical status and behaviors were constantly monitored.

### 2.6 Vaccination program and challenge

**Figure 1:**
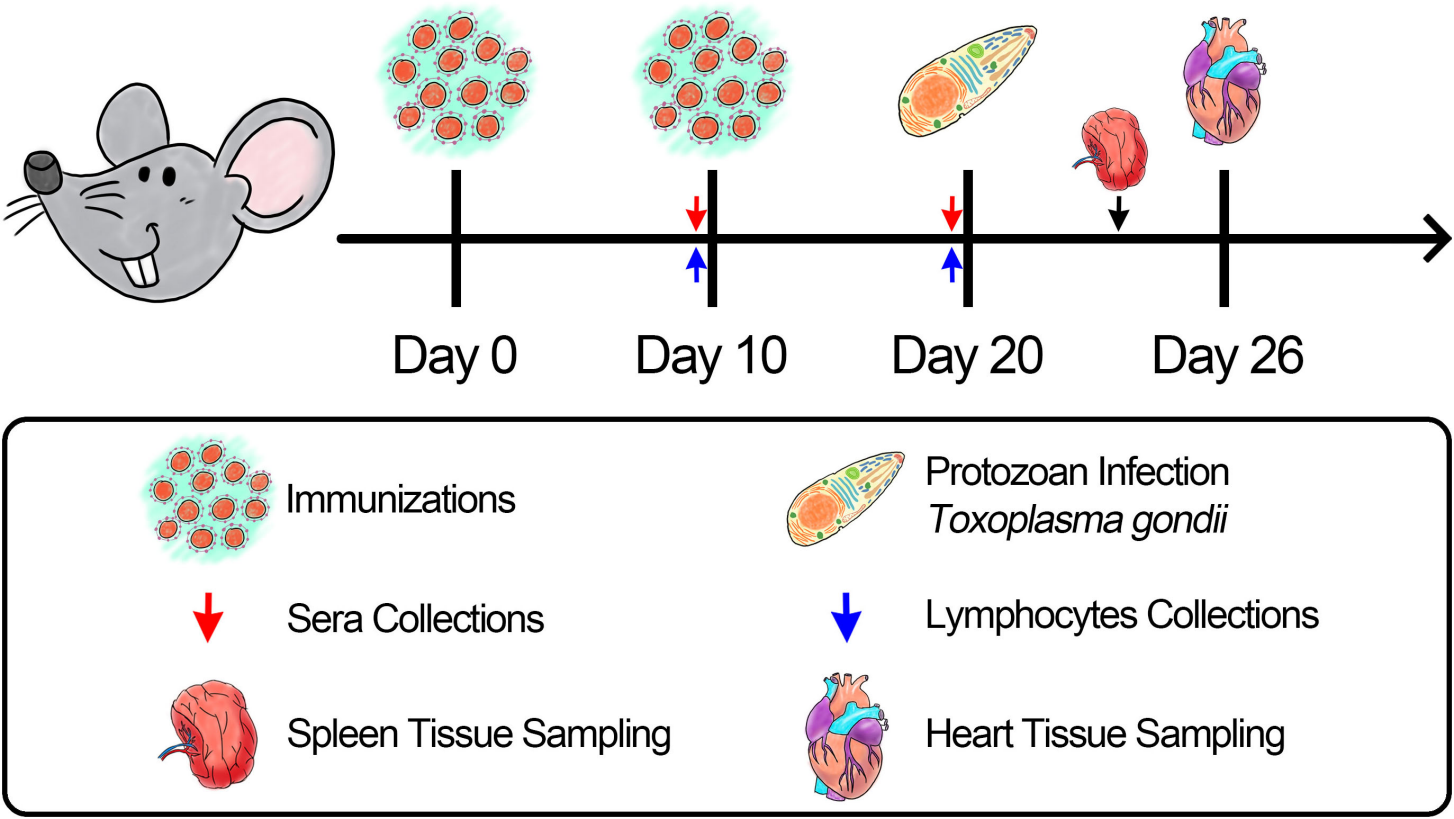
Schematic illustration of the vaccination trial with *T. gondii*.

ICR mice were randomly allocated into nine groups with 21 replicates in each group. Animals were immunized subcutaneously in the back with multipoint, and the maximum dosage for single injection was controlled within 500 μL. The booster vaccination using identical criteria was performed 14 days after the primary immunization (**Table 1**). Animals in the experimental group received an injection containing 100 μg of purified rTgGAP45, while animals in the control group received the same dosage without rTgGAP45 in the same manner. To estimate the protective efficacy generated by immunizations as described previously (4), eight animals from each group received an intraperitoneal injection with a lethal dose of RH strain (100 tachyzoites per mouse) 10 days after booster vaccination (**Figure 1**). All the animal trials were conducted under the supervision of the Animal Ethics Committee, Ningxia University, Yinchuan, China.

**Table 1:** Group assignment and vaccination program.

Group	Treatment (each mouse)	Time for vaccination	Infection dose (each mice)
Blank	Equal volume of 1 × PBS	On days 0 and 11	100 tachyzoites were challenged intraperitoneally on day 20
Control	100 μg of pET-32a vector protein		
PLGA	Equal volume of PLGA nanoparticle loading 1 × PBS		
206VG	Equal volume of Montanide™ ISA 206 VG emulsions loading 1 × PBS		
660VG	Equal volume of Montanide™ ISA 660 VG emulsions loading 1 × PBS		
TgGAP45	100 μg of rTgGAP45		
TgGAP45-PLGA	TgGAP45-PLGA nanoparticles containing 100 μg of rTgGAP45		
TgGAP45-206VG	Montanide™ ISA 206 VG emulsions containing 100 μg of rTgGAP45		
TgGAP45-660VG	Montanide™ ISA 660 VG emulsions containing 100 μg of rTgGAP45		

### 2.7 Detection of antibody and cytokine secretion

On days 10 (10 days after the first immunization) and 20 (10 days after the booster immunization), bloods were harvested from the eye sockets, and the sera were prepared. To determine the titers of *T. gondii*-specific serum antibody in the sera, enzyme-linked immunosorbent assays (ELISAs) were carried out as previously mentioned (46). In brief, each well of the 96-well plates (Biosharp, Life Science, Hefei, China) was incubated with 1 μg of *T. gondii* STAg (dissolved in 100 μL carbonate buffer pH 9.6) overnight at 4°C. After being rinsed in TBST for 5 min, each well was blocked with 100 μL of TBST containing 5% (w/v) skimmed milk power at 37°C for 1 h. After being washed in TBST for three times, plates were incubated with 100 μL of the animals’ sera (each well) for 1 h at 37°C. Plates were incubated with HRP-conjugated anti-mouse IgG (ABclonal Technology, Wuhan, China) at 1:10,000 dilutions (100 μL each well) at 37°C for 1 h. After being washed, each well was incubated with 100 μL of 3,3',5,5'-tetramethylbenzidine (TMB, Beyotime Biotech, Shanghai, China) to develop colors at room temperature, and the reaction in each well was stopped by adding 100 μL of 2 M H_2_SO_4_ solutions. Then, the absorbance at OD_450_ was investigated by a microplate photometer (Bio Tek Instruments, VT, USA) within 30 min. Each group contained five replicates, and each replication was measured once.

To further investigate the humoral immunity of immunized animals, animals’ sera collected on day 20 (10 days after the booster immunization) were conducted by commercially available ELISA kits (Enzyme-linked Biotechnology, Shanghai, China). Based on double antibody sandwich ELISA, the levels of interferon-gamma (IFN-γ), interleukin-4 (IL-4), transforming growth factor-β (TGF-β), IL-6, IL-10, and IL-17 in the animals’ sera were investigated in strict accordance with the requirements. Each group contained five replicates, and each replication was measured once.

### 2.8 Lymphocyte proliferation assay

On day 19 (1 day before the challenge), three animals from each group were euthanized to isolate splenic lymphocytes using the separation solution (Solarbio, Beijing, China) according to the instructions. The obtained lymphocytes were adjusted to 10^5^ cells/well in Dulbecco’s Modified Eagle’s Medium (DMEM, Gibco Life Technologies, Carlsbad, CA, USA) containing 20% (v/v) fetal bovine serum (FBS, Gibco Life Technologies, Carlsbad, CA, USA) and 20 μg/mL purified TgGAP45 proteins, and cultured in a 96-well plate. After a 72-h incubation at 37°C in a 5% (v/v) CO_2_ atmosphere, each well was added with 10 μL of Cell Counting Kit 8 reagent (CCK-8, Beyotime Biotech, Shanghai, China), and was sequentially cultured for 4 h. Then, the absorbance at OD_450_ was determined by a microplate photometer. Each group contained three animals, the lymphocytes from each animal were divided into three replicates, and each replication was measured once.

### 2.9 Flow cytometry analysis

On days 10 (10 days after the first immunization) and 20 (10 days after booster immunization), five animals from each group were sacrificed to harvest splenic lymphocytes by using the same separation solution described in Section 2.8, and the obtained lymphocytes were resuspended in 1 × PBS. To evaluate the surface marker of dendritic cells (DCs), lymphocytes were first incubated in DMEM media containing 10% (v/v) fetal bovine serum and 1% (w/v) double antibiotics at 37°C in a 5% (v/v) CO_2_ atmosphere overnight. Subsequently, 10^6^ attached lymphocytes were gently harvested and re-dissolved in 100 μL of 1 × PBS, and cells were stained with APC-conjugated anti-mouse CD11c, FITC-conjugated anti-mouse CD86, and PE-conjugated anti-mouse CD83 (eBioscience, San Diego, CA, USA) for 1 h at 4°C in the dark. After washing in 1 × PBS, cells were re-collected and sorted by flow cytometry (Beckman Coulter Inc, Brea, CA, USA). Before cell sorting, adequate fluorescence compensation was adjusted according to the fluorescence minus one (FMO) controls, and the gating strategies are shown in **Supplementary Figure S1**.

To analyze the MHC molecule changes in DCs, 10^6^ splenic lymphocytes were resuspended in 100 μL of 1 × PBS, stained with PE-conjugated anti-mouse CD11c, FITC-conjugated anti-mouse MHC-I, and APC-conjugated anti-mouse MHC-II (eBioscience, San Diego, CA, USA) under the same criteria, and sorted by the flow cytometry using the same strategies described previously.

As for the proportion of CD4^+^ and CD8^+^ T lymphocytes, 10^6^ splenic lymphocytes were also resuspended in 100 μL of 1 × PBS and stained with FITC-conjugated anti-mouse CD3e, PE-conjugated anti-mouse CD8, and FITC-conjugated anti-mouse CD4 (eBioscience, San Diego, CA, USA). The staining strategy and flow cytometry analysis were performed using the same strategies mentioned previously.

### 2.10 *T. gondii* burdens in animals

Indirect immunofluorescence assay (IFA) was recruited to determine *T. gondii* burdens in the spleen. In brief, on day 24 (4 days after the challenge), three animals from each group were euthanized to isolate the spleen. The obtained spleen was fixed in 10% neutral-buffered formalin, embedded in paraffin, and sliced by a tissue slicer (Leica Biosystems, Deer Park, TX, USA). After dehydration in graded ethanol and vitrification by dimethylbenzene (Macklin Biochemical Technology, Shanghai, China), sections were immersed in repair buffer (Servicebio, Wuhan, China) and incubated at 95°C for 20 min. After antigen retrieval, sections were blocked in TBST containing 5% (w/v) skimmed milk powder for 1 h at 37°C, and stained with rabbit anti-*T. gondii* (Abcam, Waltham, MA, USA) at 1:1,000 dilutions in TBST overnight at 4°C on a rotary shaker. Rinsed in TBST for three times, sections were then incubated with CY3-conjugated anti-rabbit IgG (1: 5,000 dilutions, ABclonal Technology, Wuhan, China) for 1 h at 37°C. Before sealing, sections were stained with DAPI dihydrochloride (Beyotime Biotech, Shanghai, China) for 5 min at room temperature. Finally, three visual fields were randomly chosen from one section, and visualized by Pannoramic MIDI (3DHistech Ltd, Budapest, Hungary). Each group contained three animals, and one section was made from each tissue.

To analyze the immune protective efficacy elicited by vaccinations, absolute quantitative real-time PCR (qPCR) was performed to investigate *T. gondii* burdens in the cardiac tissue of challenged animals. Five animals from each group were sacrificed to isolate the cardiac tissue on day 26 (6 days after the challenge), and 30.0 mg of collected tissue was lysed to obtain genomic DNA per the guidelines of a commercial kit (OMEGA Bio-tek, Norcross, Georgia, USA). As previously described (47), the 529-bp fragments in the extracts were amplified by ChamQ Universal SYBR qPCR MasterMix (Vazyme, Nanjing, China) using a CFX96 amplifier (Bio-Rad, Hercules, CA, USA). The reference amplification was also conducted by using the constructed plasmids as DNA template, and the melting curve analysis was also carried out at the end of amplifications to remove unexpected amplification. Each group contained 10 replications, and each replication was detected once.

### 2.11 Statistical analysis

Data analysis was conducted using GraphPad Prism 9.0 (GraphPad Software, San Diego, CA, USA). Statistical differences between two groups were evaluated by one-way analysis of variance (ANOVA) followed by Dunnett’s test. Comparisons among TgGAP45, TgGAP45-PLGA, TgGAP45-206VG, and TgGAP45-660VG groups were illustrated by ANOVA following Bonferroni’s correction. Significance was considered at *p<* 0.05, and values in all graphs were presented as mean ± standard deviation (SD).

## 3 Results

### 3.1 Expression of rTgGAP45 and Western blot analysis

**Figure 2:**
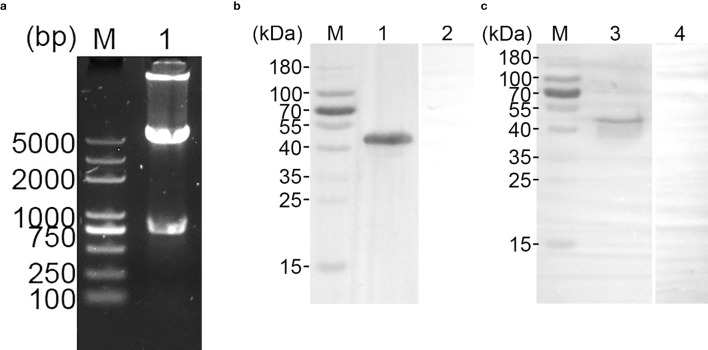
**(a)** Double digestion analysis of pET-32a-TgGAP45 plasmids. Line M: DL5,000 marker; Line 1: Double digestion of the constructed plasmid by *Bam*HI and *EcoR*I. **(b)** SDS-PAGE analysis of purified recombinant TgGAP45 proteins. Line M: MW marker proteins. Line 1: purified recombinant proteins. **(c)** Western blot analysis of purified recombinant and native TgGAP45 proteins. Purified recombinant TgGAP45 proteins were detected by sera against *T. gondii* STAg (Line 1) or PBS (Line 2), while native TgGAP45 proteins from *T. gondii* STAg were detected by sera against recombinant TgGAP45 proteins (Line 3) or PBS (Line 4). Line M: MW marker proteins.

The recombinant pET-32a-TgGAP45 plasmid was well constructed as previously described (**Supplementary Figure S2a**). Double enzyme digestion was conducted by using *Bam*HI and *Eco*RI to verify the constructed vector, yielding two fragments of 744 and 5,894 bp (**Figure 2a**). Furthermore, plasmid sequencing also indicated that the inserts of the recombinant vector were correct (**Supplementary Figure S2b**). According to the instructions of the pET-32a vector, the recombinant TgGAP45 protein generated by the constructed vector consisted of a tag protein (17.7 kDa) and TgGAP45 (27.3 kDa). Thus, the theoretical molecular weight of recombinant TgGAP45 was 45.0 kDa, which matched the obtained result shown in **Figure 2b**. By endotoxin eradication assay, the endotoxin level of purified rTgGAP45 decreased to 0.01 EU/mL. Moreover, evaluated by immunoblot analysis shown in **Figure 2c**, recombinant TgGAP45 protein could be identified by the rat’s serum against STAg, and the natural TgGAP45 could be detected by the serum against rTgGAP45, indicating a satisfactory antigenicity of rTgGAP45, which could induce the immune response in host.

### 3.2 Characteristics of synthesized nanoparticles

**Figure 3:**
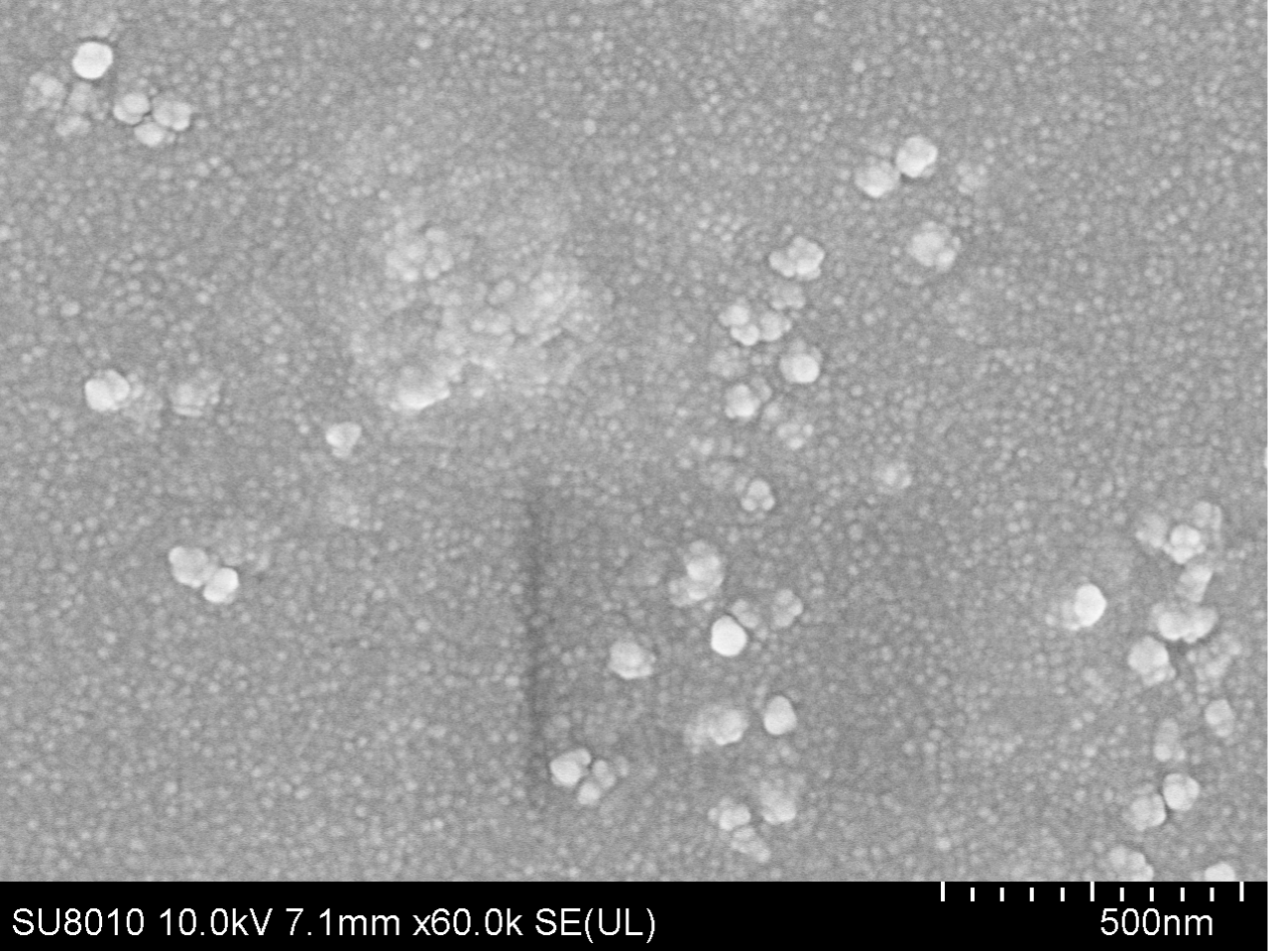
The SEM observation of PLGA nanoparticles loaded with recombinant TgGAP45 proteins. A double emulsion solvent evaporation technique was employed to formulate TgGAP45PLGA nanoparticles. Bar represented 500 nm.

To validate whether the synthesized nanoparticles were in nanoscale, the TgGAP45-PLGA nanoparticles were imaged and the results showed that TgGAP45-PLGA nanoparticles were spherical and well-proportioned with a rough surface (**Figure 3**). Based on the SEM images, the average diameter of TgGAP45-PLGA nanoparticles was determined to be 65.93 ± 3.13 nm (*n* = 10), with a PDI of 0.296 ± 0.03. Furthermore, the EE was 74.97%, according to three independent experiments.

**Figure 4:**
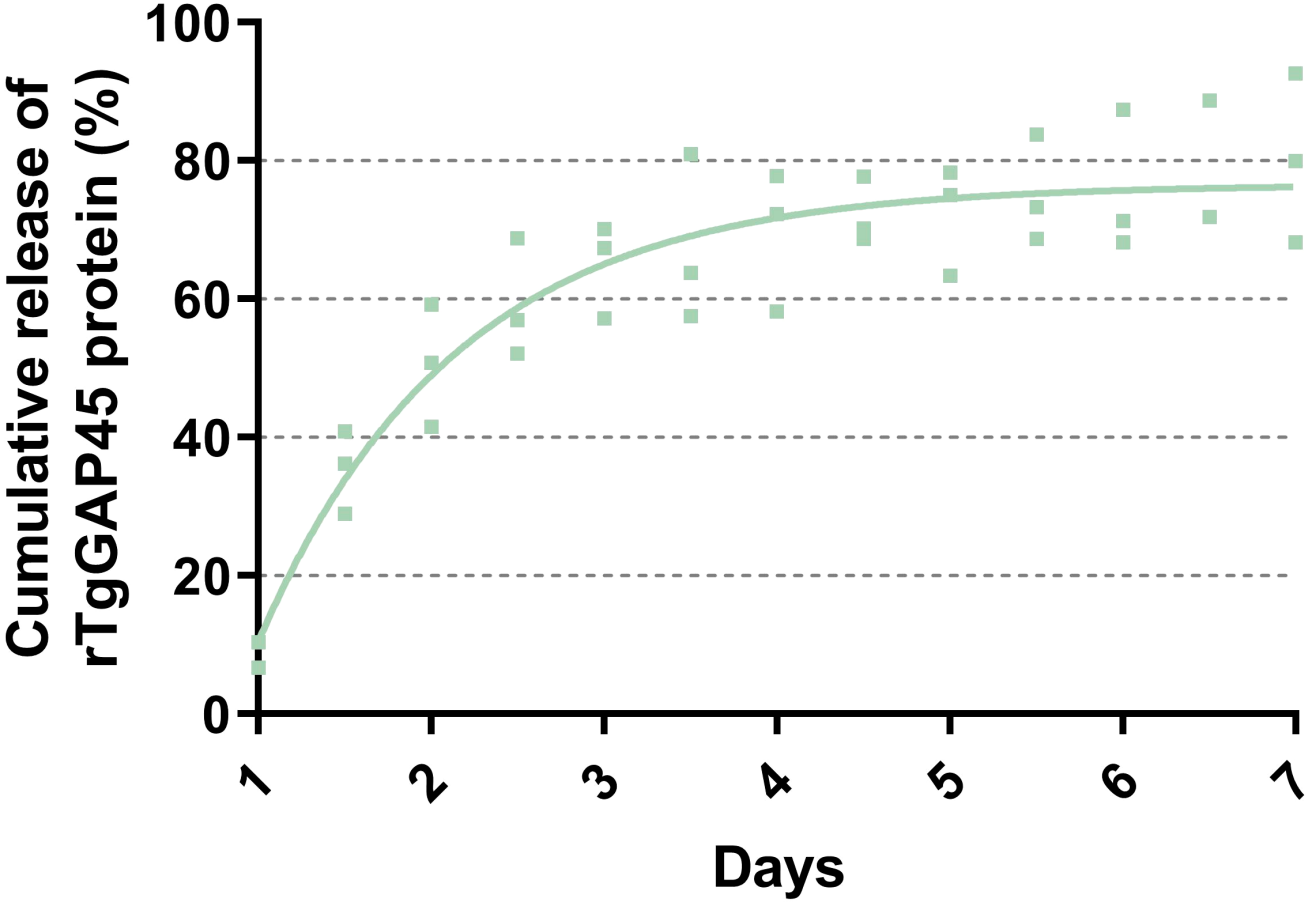
The release profile of TgGAP45-PLGA nanoparticles *in vitro* over a 7-day period. The concentrations of uncombined proteins in the supernatant were analyzed by commercial BCA kits, and the release curve was constructed by the concentrations of uncombined proteins and their total volumes. The fit line was determined by the least-square fitting method. Three independent experiments were carried out, and each sample was detected once. Values were presented as scattered points (*n* = 3), while the mean values were represented by the curve line.

Analyzed by a sustained slow release over a 1-week period, the release profile of TgGAP45PLGA was constructed. As demonstrated in **Figure 4**, a burst release with approximately 9.23% of rTgGAP45 was observed at the initial stage, and such phenomenon may be caused by the recombinant proteins coated to the surface of PLGA nanoparticles. Obtained by a fit curve constructed with a coefficient of determination (*R* ^2^) of 0.8741, similar results were also indicated. In addition, the release curve was relatively steep in the first 4 days, while the trend became smoother after the fourth day.

**Figure 5:**
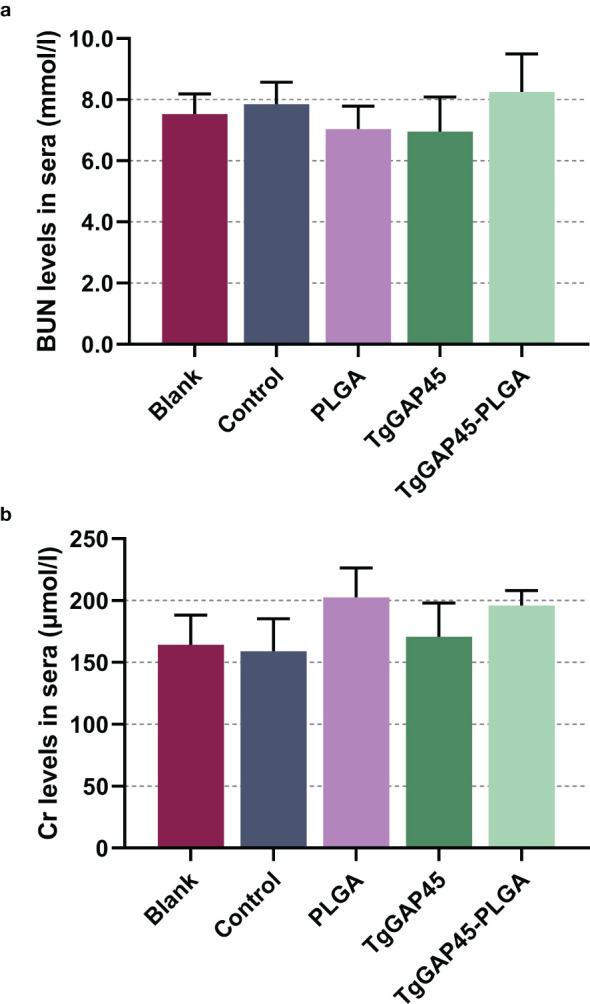
The toxicity of recombinant TgGAP45 proteins and their PLGA nanoparticles. The endotoxins in recombinant proteins were removed multiple times, and the levels of BUN **(a)** and Cr **(b)** in the animals’ sera were evaluated by the commercially available kits. Each group contains five animals, and each serum was investigated once. Values were estimated using one-way ANOVA followed by Dunnett’s test. Comparisons between TgGAP45 and TgGAP45-PLGA groups were conducted by ANOVA following Bonferroni’s correction. Values were presented as the mean of the mean ± SD (*n* = 5).

**Figure 6:**
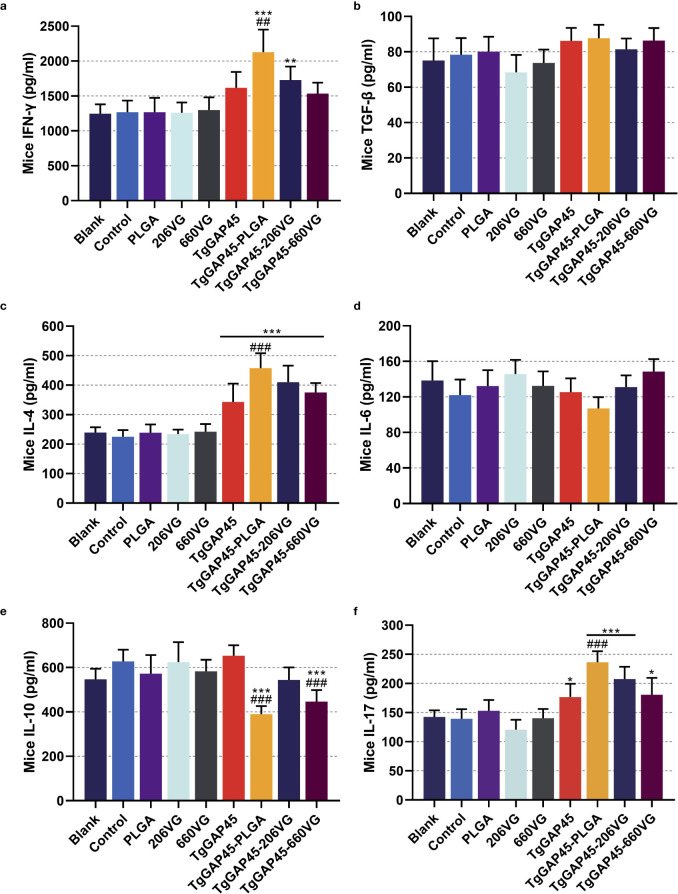
Cytokine secretions in animals’ sera. Commercially available ELISA kits were purchased to determine the concentrations of IFN-γ **(a)**, TGF-β **(b)**, IL-4 **(c)**, IL-6 **(d)**, IL-10 **(e)**, and IL-17 **(f)** in the sera on day 20 (10 days after the booster immunizations). Each group involved five replications, and each replication was detected once. Values were estimated using one-way ANOVA followed by Dunnett’s test. Comparisons among TgGAP45, TgGAP45-PLGA, TgGAP45-206G, and TgGAP45-660VG groups were conducted by ANOVA following Bonferroni’s correction. Values were presented as the mean of the mean ± SD (*n* = 5). *p< 0.05, **p< 0.01, ***p< 0.001, ##*p<* 0.01, and ###*p<* 0.001.

As a premise of *in vivo* research, the safety of nanoparticles is of major concern before application, even if it involves nontoxic nanomaterials with biodegradable characteristics. Thus, we tested the toxicity of synthesized nanoparticles in mice (**Figure 5**). The levels of BUN and Cr in animals’ sera were maintained in an acceptable level, and no significant difference occurred (*p* > 0.05) in all immunized animals, indicating that the recombinant TgGAP45 proteins and their PLGA nanoparticles could not affect the function of animals’ liver and kidney. Furthermore, the mental health of each animal was also recorded, and no adverse reaction was observed during the trials. The induction of a balanced Th1/Th2 immune response is critical for effective defense against intracellular pathogens like *T. gondii*. Our findings demonstrate that “TgGAP45-PLGA nanoparticles elicited a mixed Th1/Th2 profile,” characterized by elevated IFN-γ (Th1) and IL-4 (Th2) levels (**Figure 6c**), which aligns with the dual functionality required for parasite containment (cellular immunity) and humoral protection (antibody-mediated neutralization). All these obtained results suggested that the recombinant TgGAP45 proteins as well as their nanoparticles were nontoxic to mice.

### 3.3 Level of antibodies and cytokines

**Figure 7:**
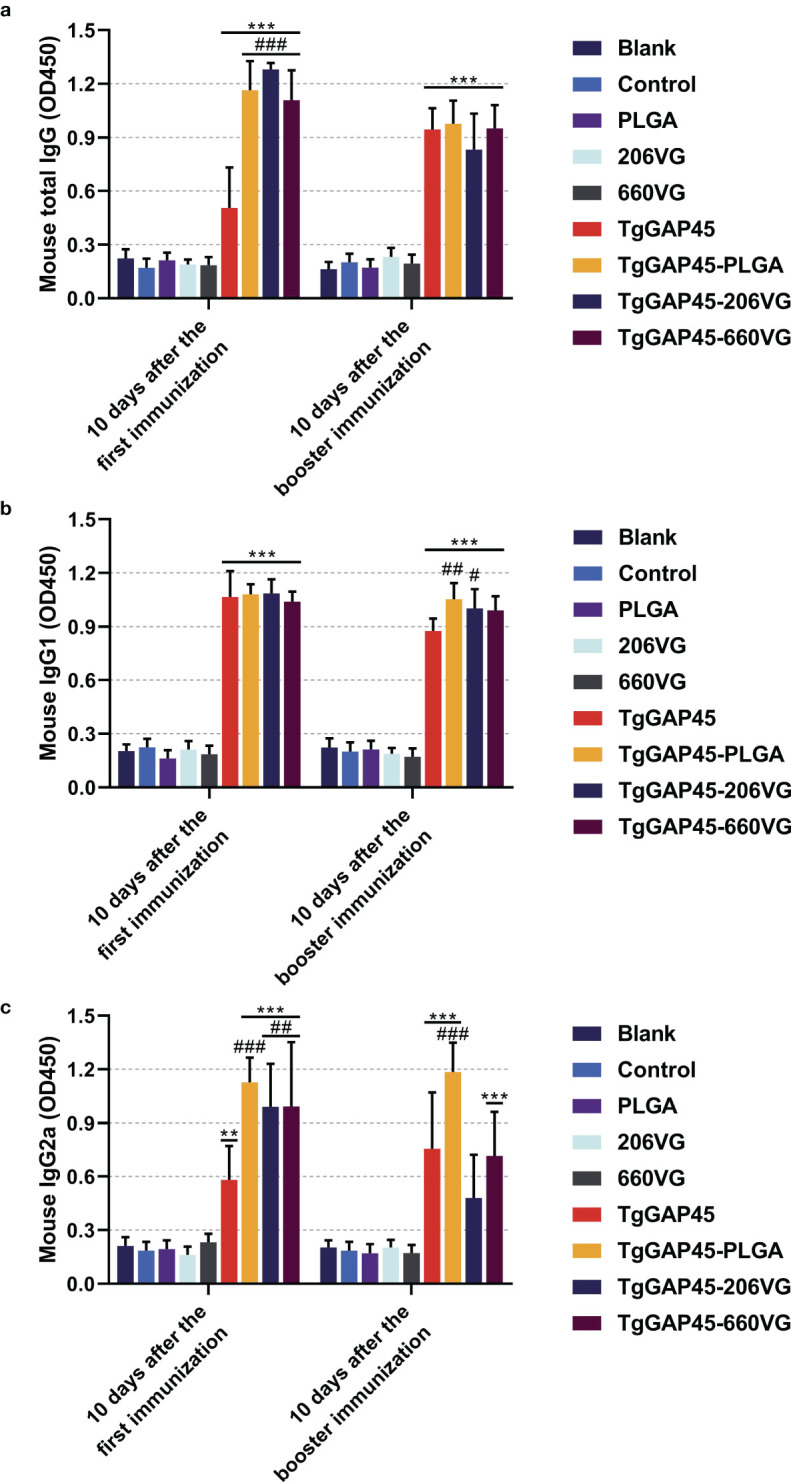
Antibody determination of total IgG **(a)** and isotypes IgG1 **(b)** and IgG2a **(c)** in animals’ sera 10 days after the first (day 10) and booster immunizations (day 20). Each group involved five replications, and each replication was detected once. Values were estimated using one-way ANOVA followed by Dunnett’s test. Comparisons among TgGAP45, TgGAP45-PLGA, TgGAP45-206G, and TgGAP45-660VG groups were conducted by ANOVA following Bonferroni’s correction. Values were presented as the mean of the mean ± SD (*n* = 5). **p< 0.01, ***p< 0.001, #*p<* 0.05, ##*p<* 0.01, and ###*p<* 0.001.

To determine the levels of IgG, IgG1, and IgG2a induced by nanoparticles and two types of emulsions, animals were immunized according to the group assignment and vaccination program mentioned in **Table 1**, and sera were collected from all immunized mice. As shown in **Figure 7**, animals immunized with TgGAP45 as well as its preparations could remarkably induce higher levels of total IgG and IgG1 (*p*< 0.001), while naked proteins and its preparations could equally enhance the release of IgG2a after the first immunization (*p*< 0.01). As for the isotypes illustrated in **Figures 7b, c**, TgGAP45-PLGA nanoparticles could significantly promote the generation of IgG1 and Ig2a compared with the naked proteins (*p*< 0.01). Compared with the TgGAP45 group, animals in the TgGAP45-206VG group could elicit statistically higher levels of IgG1 after the booster immunization (*p*< 0.05), while both tested emulsions could increase the generation of IgG2a after the first immunization (*p*< 0.01).

To further analyze humoral immunity in vaccinated animals, sera were collected from each group and analyzed by the commercially available ELISA kits. As for the Th1 immune response exhibited in **Figures 6a, c**, TgGAP45-PLGA nanoparticles could enhance the secretion of IFN-γ and IL-4 when compared with the control or naked TgGAP45 group (*p*< 0.01). Only TgGAP45-206VG emulsions could both stimulate the generations of IFN-γ and IL-4 when compared with the control group (*p*< 0.01). As for the Th2 immune response demonstrated in **Figures 6b, e**, TgGAP45-PLGA nanoparticles and TgGAP45-206VG emulsions could inhibit the secretion of IL-10 when compared with control or recombinant proteins (*p*< 0.001). In addition, no significant difference was detected in all prepared agents in eliciting TGF-β (*p* > 0.05). Regarding the Th17 immune response demonstrated in **Figure 6f**, all nanoparticles and emulsions could promote higher levels of IL-17 than the controls in animals’ sera (*p*< 0.05), while only TgGAP45-PLGA nanoparticles could generate higher IL-17 when compared with the naked proteins (*p*< 0.001). Regarding the inflammatory cytokine displayed in **Figure 6d**, all prepared agents containing recombinant TgGAP45 proteins generated similar effects in eliciting IL-17 in mice (*p* > 0.05).

### 3.4 Maturation and differentiation of DCs in immunized mice

**Figure 8:**
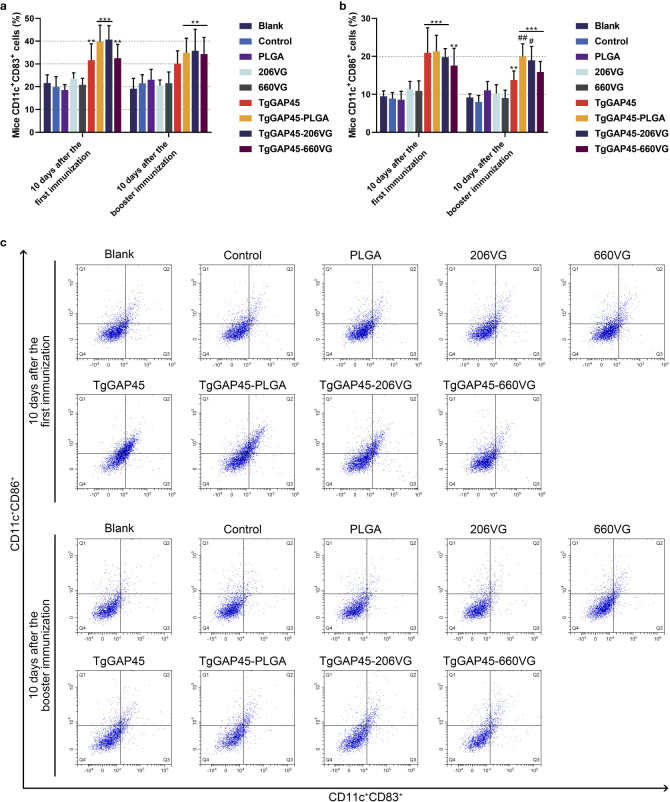
Flow cytometry analysis on the maturation of DCs in the splenic cells isolated from the immunized animals. Five animals in each group were sacrificed and splenic cells from each animal were harvested and investigated. The bar graph showed the ratio of CD83 **(a)** and CD86 molecules **(b)** on the surfaces of splenic DCs, while the dot plots **(c)** showed the percentages of CD11c^+^CD83^+^ and CD11c^+^CD86^+^ cells. Each group involved five replications, and each replication was detected once. Values were estimated using one-way ANOVA followed by Dunnett’s test. Comparisons among TgGAP45, TgGAP45-PLGA, TgGAP45-206G, and TgGAP45-660VG groups were conducted by ANOVA following Bonferroni’s correction. Values were presented as the mean of the mean ± SD (*n* = 5). **p< 0.01, ***p< 0.001, #*p<* 0.05, and ##*p<* 0.01.

On days 10 (10 days after the first immunization) and 20 (10 days after booster immunization), five mice from each group were sacrificed to harvest splenic lymphocytes to evaluate the maturation of DCs by flow cytometry. As illustrated in **Figures 8a–c**, the synthesized nanoparticles as well as the prepared emulsions could significantly induce the expressions of CD83 and CD86 molecules in splenic DCs when compared with the controls (*p*< 0.01). However, compared with the naked proteins, only TgGAP45-PLGA nanoparticles and TgGAP45-206VG emulsions could enhance the proportions of CD11c^+^CD86^+^ cells in the spleens (*p*< 0.05).

**Figure 9:**
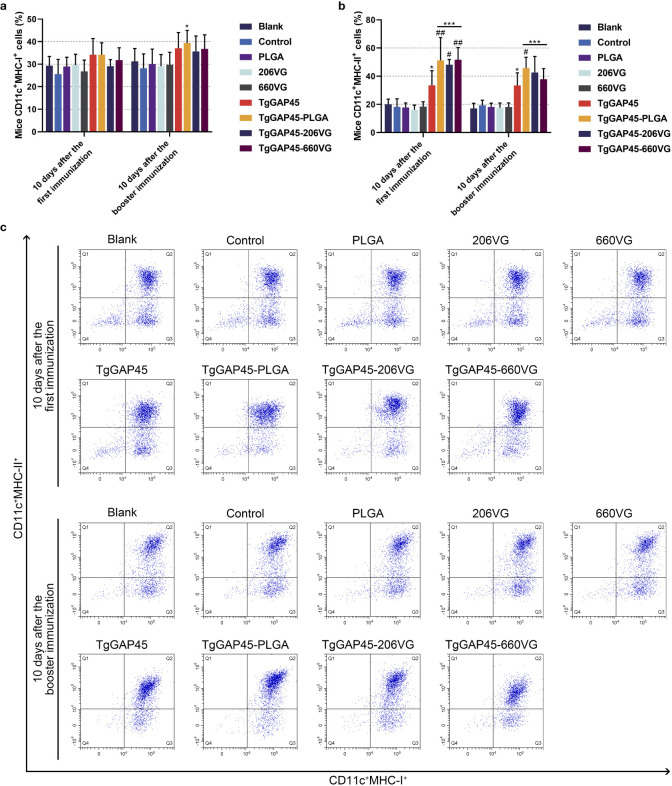
The investigation of MHC molecules on the surface of splenic DCs harvested from immunized animals. Five animals in each group were sacrificed and splenic cells from each animal were harvested and analyzed. The bar graph showed the ratio of MHC-I **(a)** and MHC-II molecules **(b)** on the surfaces of splenic DCs, while the dot plots **(c)** showed the percentages of CD11c^+^ MHCI^+^ and CD11c^+^ MHC-II^+^ cells. Each group involved five replications, and each replication was detected once. Values were estimated using one-way ANOVA followed by Dunnett’s test. Comparisons among TgGAP45, TgGAP45-PLGA, TgGAP45-206G, and TgGAP45-660VG groups were conducted by ANOVA following Bonferroni’s correction. Values were presented as the mean of the mean ± SD (*n* = 5). *p< 0.05, ***p< 0.001, #*p<* 0.05, and ##*p<* 0.01.

Mature DCs would differentiate and present antigens. Flow cytometry was conducted to analyze the MHC molecule changes in DCs isolated from immunized mice. As shown in **Figures 9a, c**, only animals in the TgGAP45-PLGA group were detected with higher expressions of MHC-I molecules when compared with those in the control group (*p*< 0.05). As for the MHC-II molecules displayed in **Figures 9b, c**, all agents containing rTgGAP45 could significantly promote its expressions in CD11c^+^ cells (*p*< 0.05). Moreover, mice in TgGAP45-PLGA nanoparticles could elicit higher proportions of CD11c^+^MHC-II^+^ cells than those in the TgGAP45 group (*p*< 0.05), while two types of emulsions, after the first immunization, could only enhance the percentage of CD11c^+^MHC-II^+^ cells when compared with the naked proteins (*p*< 0.05).

### 3.5 Determination of lymphocyte proliferation

**Figure 10:**
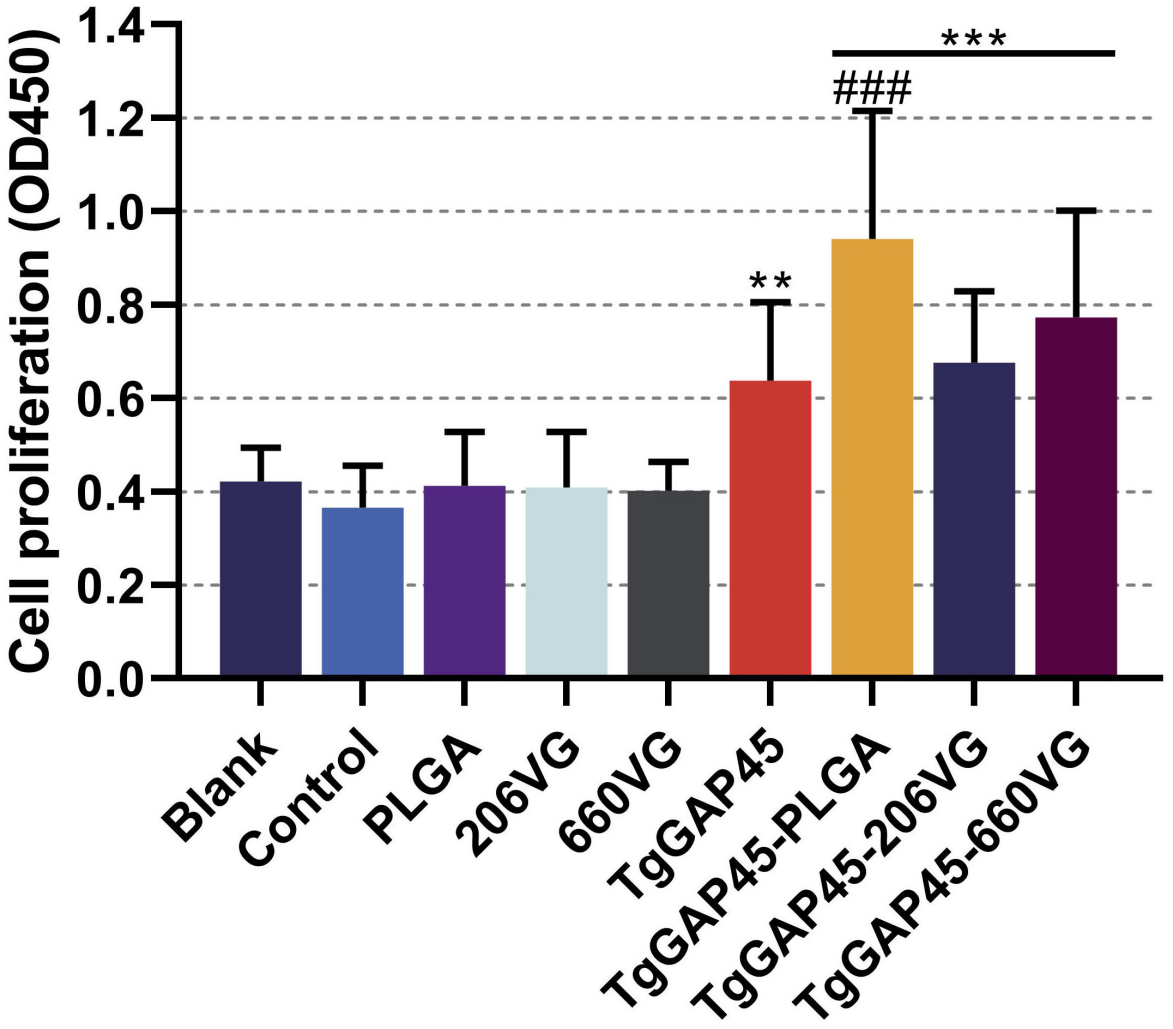
Splenocyte proliferation of immunized mice. Three mice in each group were sacrificed and the lymphocytes from each mouse were collected, the obtained lymphocytes were then divided into three parts, and each part of the lymphocyte was cultured with 10 μg/mL rTgGAP45 protein. Each part of the lymphocytes was investigated once, and values were estimated using one-way ANOVA followed by Dunnett’s test. Comparisons among TgGAP45, TgGAP45-PLGA, TgGAP45-206G, and TgGAP45-660VG groups were conducted by ANOVA following Bonferroni’s correction. Values were presented as the mean of the mean ± SD (*n* = 9). **p< 0.01, ***p< 0.001, and ###*p<* 0.001.

On day 19 (1 day before the challenge), three mice from each group were euthanized to isolate splenic lymphocytes. Then, the effects of TgGAP45 proteins as well as its nanoparticles and emulsions in promoting T-cell proliferation were investigated by using a commercial CCK-8 reagent. As shown in **Figure 10**, vaccinations with TgGAP45 and its nanoparticles and emulsions could significantly enhance lymphocyte proliferation when compared with the controls (*p*< 0.01).

Furthermore, TgGAP45-PLGA nanoparticles generated the ability to enhance cell proliferation when compared with the naked proteins (*p*< 0.001).

### 3.6 Identification of CD4^+^ and CD8^+^ T lymphocyte proportions

**Figure 11:**
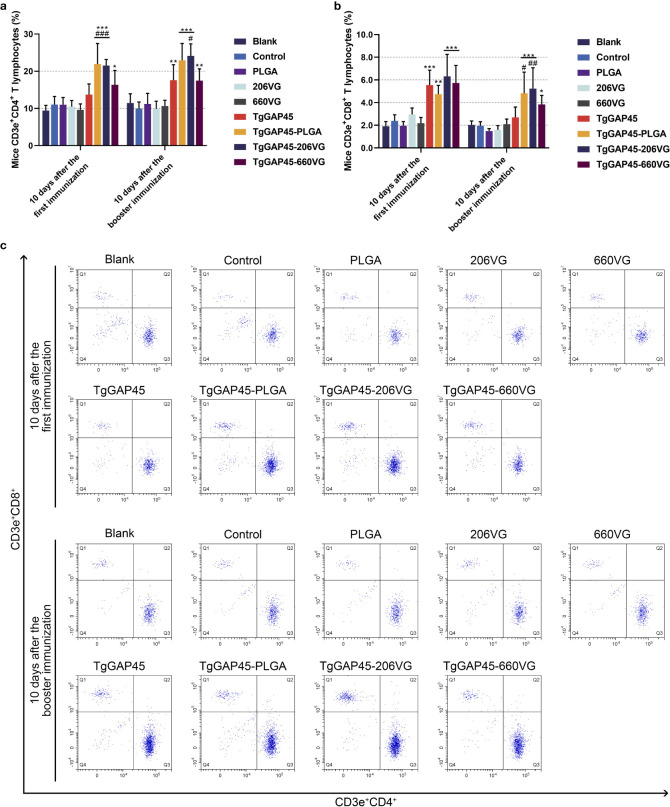
The proportions of T lymphocytes in animals’ spleen. Five animals in each group were sacrificed and splenic cells from each animal were harvested and analyzed. The bar graph showed the ratio of CD4^+^ **(a)** and CD8^+^ T lymphocytes **(b)**, while the dot plots **(c)** showed the percentages of CD3e^+^ CD4^+^ and CD3e^+^ CD8^+^ cells. Each group involved five replications, and each replication was detected once. Values were estimated using one-way ANOVA followed by Dunnett’s test. Comparisons among TgGAP45, TgGAP45-PLGA, TgGAP45-206G, and TgGAP45-660VG groups were conducted by ANOVA following Bonferroni’s correction. Values were presented as the mean of the mean ± SD (*n* = 5). *p< 0.05, **p< 0.01, ***p< 0.001, #*p<* 0.05, ##*p<* 0.01, and ###*p<* 0.001.

Ten days after the first (day 10) and the booster immunization (day 20), lymphocytes were separated by the commercial separation solution and sorted by flow cytometry. As evaluated in **Figures 11a, c**, PLGA nanoparticles as well as two types of emulsions could remarkably enhance the proportions of CD4^+^ T lymphocytes in animals’ spleens (*p*< 0.05). When compared with rTgGAP45, both TgGAP45-PLGA nanoparticles and TgGAP45-206VG emulsions exhibited a capability in enhancing the proportions of CD3e^+^CD4^+^ cells. As for the CD8^+^ T lymphocytes demonstrated in **Figures 11b, c**, three types of vaccines could notably upregulate the proportions of CD8^+^ T lymphocytes (*p*< 0.05), while TgGAP45-PLGA nanoparticles and TgGAP45-206VG emulsions could enhance the proportions of CD8^+^ T lymphocytes higher than rTgGAP45 itself after the second immunization (*p*< 0.05).

### 3.7 Indirect immunofluorescence of spleen tissue

**Figure 12:**
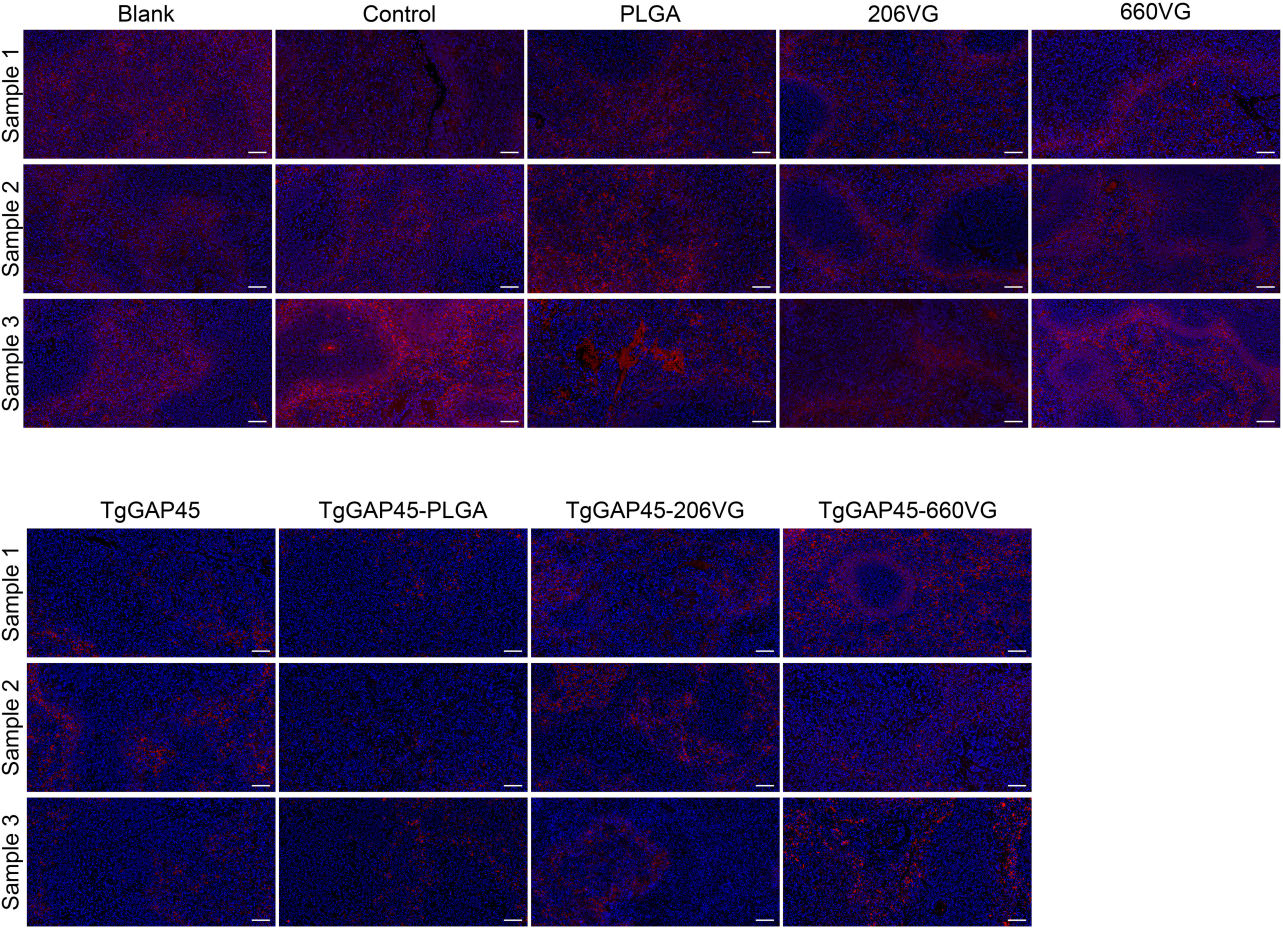
*T. gondii* burdens in animals’ spleens. Three animals were euthanized 4 days after the challenge (day 24), and the spleen tissues were harvested and then made into paraffin sections. Parasites were stained with CY3, while the nucleus of splenic cells was stained with DAPI. Each group contained three replications, and one randomly selected horizon from each section was imaged (*n* = 3). Bar represented 100 μm.

All groups of mice were challenged intraperitoneally with 100 tachyzoites on day 20 (10 days after the booster immunizations). Four days later (day 24), three randomly selected animals from each group were euthanized to isolate the spleen tissues, and the indirect IFA was carried out to analyze *T. gondii* burdens. As demonstrated in **Figure 12**, animals immunized with TgGAP45 and its preparations were detected with less parasite burdens in the spleen tissues, which suggested that three types of preparations could provide partial resistance against *T. gondii* to some extent.

### 3.8 *T. gondii* burdens in cardiac tissues

**Figure 13:**
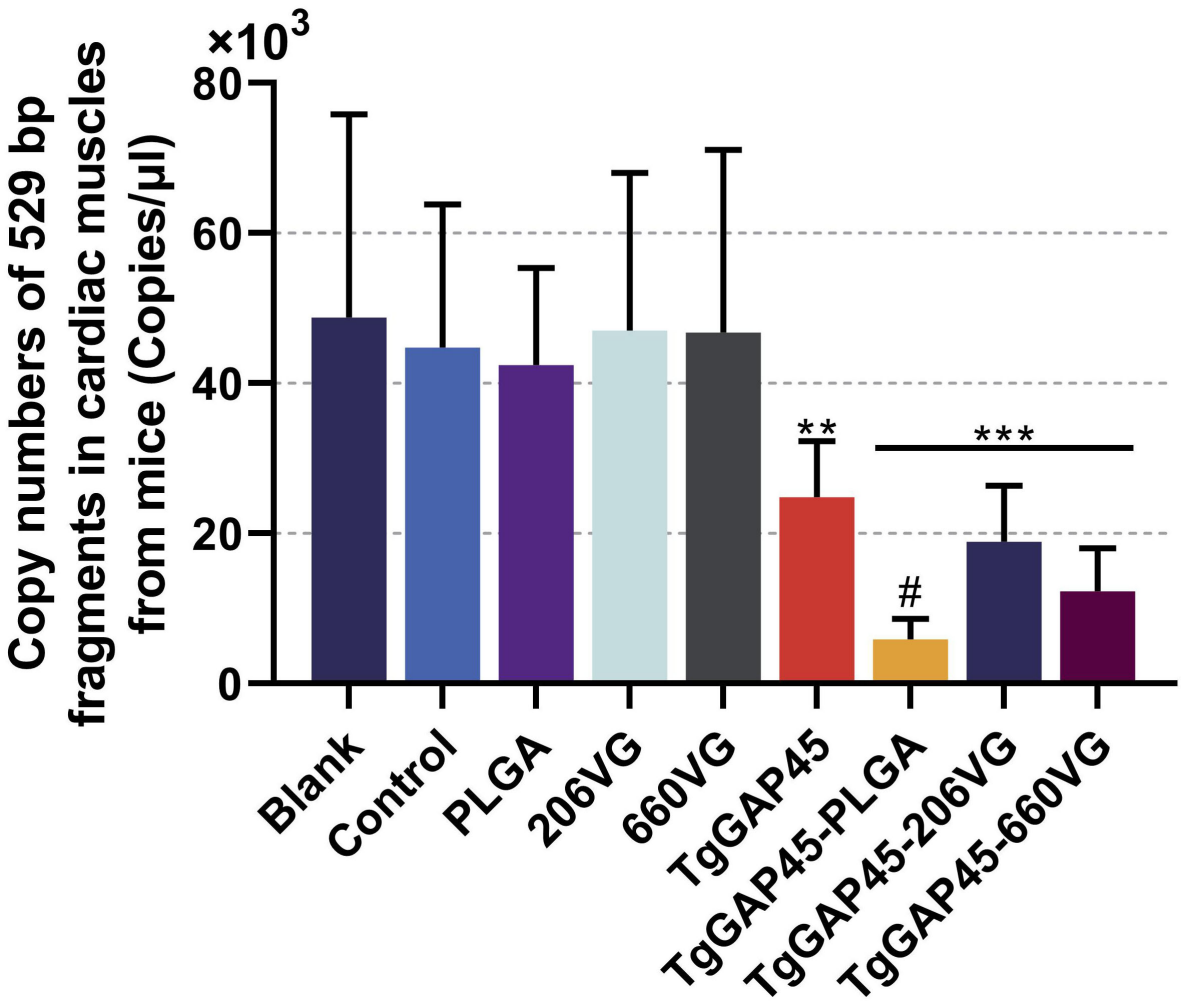
*T. gondii* burdens in animals’ cardiac muscles. Five animals were euthanized 6 days after the challenge (day 26), the cardiac muscles were collected, and the copy numbers of *T. gondii* 529-bp fragments were conducted by the absolute quantitative PCR. Each group involved five replications, and each replication was detected three times. Values were estimated using one-way ANOVA followed by Dunnett’s test. Comparisons among TgGAP45, TgGAP45-PLGA, TgGAP45-206G, and TgGAP45-660VG groups were conducted by ANOVA following Bonferroni’s correction. Values were presented as the mean of the mean ± SD (*n* = 15). **p< 0.01, ***p< 0.001, and #*p<* 0.05.

To further investigate the parasite burdens in immunized animals, five mice were euthanized 6 days after the challenge (day 26), and the cardiac tissues were collected. Then, qPCR was performed to analyze *T. gondii* burdens in the cardiac tissues of challenged animals. Compared with controls, the parasite burdens in TgGAP45, TgGAP45-PLGA, TgGAP45-206VG, and TgGAP45-660VG groups were respectively reduced by 44.45%, 86.76%, 57.80%, and 72.47% (**Figure 13**), and naked TgGAP45 proteins as well as their preparations could notably reduce the parasite burdens in cardiac tissues when compared with controls (*p*< 0.01). In addition, in comparison with recombinant TgGAP45 proteins alone, TgGAP45-PLGA nanoparticles could remarkably decrease the replications of *T. gondii* in immunized animals (*p*< 0.05).

## 4 Discussion

*T. gondii*, as a zoonotic parasitic disease, has been a threat to human and animal health for nearly a hundred years (33). However, there is no known cure or vaccine against *T. gondii* infection (48). In comparison with medical treatment, animal vaccination has been thought to be inexpensive and efficient in reducing the occurrence of toxoplasmosis (33). In recent years, minimal components from *Toxoplasma* sp. have been used as an efficient approach to induce the specific resistance. Numerous innovative vaccines, such as nucleic acid vaccines, subunit vaccines, and genetically engineered live vaccines, are constructed and proved to be effective in toxoplasmosis (49). Interestingly, there remain numerous problems in applying vaccines against toxoplasmosis, which impedes vaccine development. In this study, we demonstrated that recombinant TgGAP45 proteins prepared with PLGA nanoparticles could be used as an approach against parasitic infections. In comparison with traditional vaccines displayed in the current research, we synthesized nanoparticles that were nontoxic to animals, exhibiting satisfactory capability in eliciting humoral and cellular immunity, as well as inhibiting *T. gondii* burdens in mice. All in all, our results suggest that the enhanced activity of the antigen delivery by PLGA nanomaterials for host immunity represents a potential approach for treating toxoplasmosis.

Equipped with the enhancement of immunogenicity, adjuvants, especially aluminum salts, oil-in-water emulsions, and water-in-oil emulsions, can generate rapid, high, and long-lasting immune responses (50). However, some adjuvants potentially threaten human health due to their components, hence resulting in their unavailability in food animals (51). Currently, many studies have reported the many advantages of the application of nanomaterials in vaccine formulation, and nanomaterial-based vaccines are deemed to be highly effective and safe alternatives to traditional vaccines (52). Against such background, we prepared TgGAP45-PLGA nanoparticles based on the method of double emulsion solvent evaporation. The size of nanoparticles is closely related to the type of immune response and is a key factor determining the rate of antigen release (53, 54). Research has shown that the optimal size for lymphatic uptake is between 10 and 100 nm (55). According to the SEM images, the prepared nanoparticles were nano-sized with a rough surface, and such characteristic was regarded as easier to be absorbed by cells (56). Manolova et al. reported that nanoparticles ranging from 20 to 200 nm can freely drain into lymph nodes (LNs), and only small nanoparticles can specifically target LN-resident cells (55). According to another study, the nanoparticles with a diameter of approximately 100 nm can cross biological membranes easier than those sized approximately 1,000 nm in diameter (57). The 65.93-nm diameter of our PLGA nanoparticles (**Figure 3**) falls within the optimal range (20–200 nm) for lymphatic drainage and DC uptake, facilitating antigen trafficking to lymphoid tissues. Smaller nanoparticles (<100 nm) preferentially target LN-resident DCs, promoting MHC-I cross-presentation and CD8^+^ T-cell activation (57), consistent with our observed MHC-I upregulation in DCs (**Figure 9a**). Furthermore, the PDI observed in our study suggests a monodisperse nanoparticle population, which is critical for consistent antigen release kinetics and predictable biodistribution (58). This low polydispersity likely contributed to the observed sustained release profile and enhanced immune response by ensuring uniform nanoparticle–cell interactions (59, 60).

The sustained antigen release profile (**Figure 4**)—an initial burst (9.23% release within 12 h) followed by a gradual release over 7 days—ensures prolonged DC stimulation, critical for maintaining effector T-cell populations. This biphasic release pattern likely contributed to the robust IgG2a and IgG1 responses (**Figures 7b, c**), as persistent antigen availability supports both follicular helper T cell (Tfh)-dependent B-cell maturation and Th1/Th17 differentiation. PLGA’s anionic surface typically promotes opsonization and macrophage uptake (61), potentially enhancing antigen processing and cytokine milieu formation. The EE (74.97%) ensured sufficient antigen payload delivery, while the biodegradable PLGA matrix prevented inflammatory toxicity, as evidenced by unchanged IL-6 levels (**Figure 6d**) and normal renal markers (**Figure 5**). All these reports suggest that our synthesized nanoparticles may gain more advantages in eliciting immune response.

Nanoparticles have been proven to be effective in prolonging the bioavailability of antigens, delivering antigens to specific locations, even stimulating immune responses (62, 63). Also, the controlled release profiles are often observed from PLGA-based nanoparticles, irrespective of their size and geometry (64, 65). The slow released antigens from PLGA nanoparticles can increase the time of immunity and enhance the ability of antigen presentations in antigen-presenting cells (APCs) (59, 66). In the current research, the slow-release curve of TgGAP45-PLGA nanoparticles was constructed, and cumulative release curve of PLGA nanoparticles was steep in the first 3 days, indicating that the synthesized nanoparticles gained a significant slow-release characteristic. However, the release curve goes plane after the fourth day, and such property may be related to the full dissolution of loaded antigens. Based on the existing report, the release profile is dependent on many factors, such as the molecular weight of nanomaterials, the diameter of nanoparticles, and even the characteristic of encapsulated antigens (67).

As a nano-polymer with biocompatibility and biodegradability, PLGA has been approved by FDA for its application in clinical medicine due to its security-related features (68). Therefore, in this study, recombinant TgGAP45 proteins were encapsulated in PLGA nanomaterials to formulate TgGAP45-PLGA nanoparticles. Qualified nanoparticles should be nontoxic to animals (69); thus, the endotoxins of recombinant proteins were removed multiple times until the endotoxin levels fall to 0.01 EU/mL. In addition, only DCM was regarded as the harmful component in all reagents during the preparation and must be fully removed by evaporation (70). Thus, the TgGAP45-PLGA nanoparticles were thoroughly evaporated at room temperature and were fully freeze-fried. Subsequently, the toxicity trials were conducted *in vivo* to further analyze the toxicity of prepared nanoparticles. Undoubtedly, no toxic reaction occurred and animals were kept in good mental status, suggesting that the TgGAP45-PLGA nanoparticles were nontoxic.

Humoral immunity exhibits an essential role in fighting *T. gondii* infection, and immunoglobulin G and its subtypes have important neutralization and opsonization functions (71) and even activate the classical complement pathway to have a strong protective effect (72). In this study, massively generated IgG was identified in mice vaccinated with naked proteins and three types of preparation. In addition, subtype IgG1 antibody is relevant with Th2-type response, while the Th1-type response is associated with subtype IgG2a (73). The levels of IgG2a induced by TgGAP45-PLGA nanoparticles were slightly higher than those of IgG1, while two emulsions also showed similar results, indicating the generation of a mixed Th1/Th2-type response generated by three types of preparation.

Numerous secreted Th1-related cytokines generated an efficient role in fighting parasite infection (74), and IFN-γ is considered as the main regulatory factor of cell-mediated immunity against *T. gondii* (72). By mediating nitric oxide (NO), immune-related GTPases (IRGs), and tryptophan degradation pattern (75, 76), IFN-γ can stimulate a robust cellular immunity in higher organisms to eradicate parasites (77, 78). In the current study, mice vaccinated with TgGAP45-PLGA nanoparticles were detected with higher levels of IFN-γ, suggesting that the IFN-γ-induced Th1 immunity against *T. gondii* was activated. Furthermore, the Th2-related cytokines (IL-4 and IL-10) were also mediated by the synthesized nanoparticles. Cytokine IL-4 can enhance the secretion of IFN-γ and generate important supplementary effects on the last stage of *T. gondii* infections (79), while IL-10 can downregulate severe immunopathology activated by T lymphocyte and reduce excessive inflammatory responses to protect animals (80). Mice immunized with TgGAP45PLGA nanoparticles generated higher levels of IL-4, but lower levels of IL-10, indicating the mediations of Th2-type response. Exhibiting a dual role in toxoplasmosis, TGF-β is another key regulatory cytokine that plays a dual role in toxoplasmosis: it enhances T lymphocyte proliferation while also facilitating immune evasion through either activating apoptosis in immune cells or inducing anti-inflammatory cytokines (81). No significant difference was detected in animals immunized with either nanoparticles or two types of emulsions, revealing that TGF-β-related immunity may not be motivated by the vaccines. As a pleiotropic pro-inflammatory cytokine, IL-6 is involved in chronic inflammation as well as autoimmune diseases and is often used to indicate the occurrence of inflammation (82). The levels of IL-6 in vaccinated animals were detected as unchanged, indicating that the TgGAP45-PLGA nanoparticles and the emulsions cannot trigger inflammation during the vaccination. Secreted by Th17 cells, cytokine IL-17 is characterized by diverse biological functions, driving inflammatory responses, enhancing immune protection against pathogens, and so on (83). Currently, many reports have identified its positive effects in *T. gondii* resistance (84, 85). Such effects were also identified in the current study, and the TgGAP45-PLGA nanoparticles, as well as the TgGAP45-206VG emulsions, could promote higher levels of IL-17, indicating that the Th17-type response could be activated.

As the powerful and versatile APCs of the immune system, DCs play an important role in the activation and maintenance of host immunity under steady-state conditions (86, 87). CD83 is a critical surface marker of fully mature dendritic cells (DCs), which functionally orchestrate immune responses by activating T cell-mediated immunity (88, 89). CD86, as a costimulatory molecule, had an indispensable role in cellular binding between DCs and T lymphocytes (90). As previously reported, CD86 molecules located on the surface of DCs can bind to CD28 molecules of T lymphocytes and decrease the induction thresholds of naïve T cells (91). In this study, we explored the proportion of CD83 and CD86 molecules in vaccinated animals through flow cytometry, and the results showed that the CD83 and CD86 molecules were significantly promoted by the prepared nanoparticles and emulsions. During the activation of DCs, several-fold surface expression of MHC molecules would be detected, and these molecules can further activate CD4^+^ T lymphocytes (92, 93). Thus, MHC molecules were also investigated in the current research, and three types of vaccines could enhance the expressions of MHC-II molecules. In addition, enhanced stimulations of MHC-I molecules were only evaluated in animals immunized with TgGAP45-PLGA nanoparticles, indicating that the endogenous antigen presentation was activated. As previously reported, mature DCs with high levels of MHC-II molecules could result in the activation of CD4^+^ T cells (94), while those DCs with high levels of MHC-I molecules are associated with the induction of CD8^+^ T cells (95). Moreover, a previous study proved that the promoted expression of MHCI molecules may be related to the high release of IFN-γ (96). In any case, TgGAP45-PLGA nanoparticles and two types of emulsions could promote the activation and maturation of DCs.

Mature DCs migrate to secondary lymphoid tissues and interact with naïve T cells, finally leading to the activation of T lymphocytes (97, 98). The induced lymphocytes will experience proliferation and differentiation, and the proliferation of T lymphocytes was regarded as a main feature in evaluating the status of host immunity (99). Based on the obtained results, nanoparticles as well as two types of emulsions could significantly upregulate the proliferation of T lymphocytes, especially the synthesized nanoparticles. In addition, the type of immune responses will be determined by the differentiation of T lymphocytes (100). Based on previous reports, activated CD4 T lymphocytes can regulate host immunity by regulating cytokine production as well as macrophage recruitment and activation (101, 102), while the activated CD8 T lymphocytes can further differentiate into cytotoxic T lymphocytes (CTLs) and exhibit immunotoxicity to parasites (103). In our research, vaccinated mice generated higher proportions of CD4 and CD8 T lymphocytes, suggesting a critical role in enhancing the proportions of CD4 and CD8 T lymphocytes during *T. gondii* immunizations.

To investigate the protective efficacy of the TgGAP45-PLGA nanoparticles against the acute phase of toxoplasmosis, the animals were challenged with 100 tachyzoites on day 20 (10 days after the booster vaccination), and the *T. gondii* burdens in heart tissue were analyzed by quantitative PCR. Nanoparticle-immunized mice received the lowest burdens, suggesting that TgGAP45-PLGA nanoparticles could provide immunoprotection. In addition, similar results were also observed in spleen tissues. At present, therapeutics that could offer fully immune protection against *T. gondii* infection are still unavailable (14, 104), and our synthesized nanoparticles encapsulated with TgGAP45 may illuminate the path for upcoming vaccines.

In this study, the protective efficacy of TgGAP45-PLGA, TgGAP45-206VG, and TgGAP45660VG was evaluated by immunizing ICR mice according to the immunization table. The research results suggested that TgGAP45-PLGA, TgGAP45-206VG, and TgGAP45-660VG could generate high levels of IgG to induce humoral immunity, regulate the cytokines, activate DCs, and promote antigen presentation. In addition, absolute quantitative real-time PCR and indirect IFA implied that vaccinations with TgGAP45-PLGA nanoparticles could significantly reduce the replications of cardiac and spleen tissues. Compared with TgGAP45206VG and TgGAP45-660VG vaccines, PLGA nanoparticles carrying rTgGAP45 were more effective in inducing splenic lymphocyte proliferation and increasing the proportion of CD4^+^ and CD8^+^ T lymphocytes. All these obtained results demonstrated that three vaccines provided satisfactory immunoprotective effects in fighting *T. gondii*, especially the TgGAP45-PLGA nanovaccine, which shows great potential as a preventive agent for acute toxoplasmosis.

## 5 Conclusion

Collectively, our research indicated that nanoparticles loaded with recombinant TgGAP45 proteins were essential in inducing intense immunity against toxoplasmosis. Immunoprotective trials indicated that nanoparticles, compared with the traditional emulsions, could induce strong humoral and cellular responses and statistically eliminate parasites in immunized animals. All these results suggested that nanomaterials were necessary for developing an effective vaccine. In addition, our synthesized TgGAP45-PLGA nanoparticles are not perfect as we thought. Owing to the intracellular nature of *Toxoplasma* sp., further investigations should optimize the index of antigen loadings, further enhance cellular immunity, and prove its effectiveness in other susceptible animals.

## Data Availability

The original contributions presented in the study are included in the article/**Supplementary Material**. Further inquiries can be directed to the corresponding authors.

## Supplementary material

Supplementary Figure 1 Gating strategies for flow cytometry presented in the main article. Before cell sorting, adequate compensation was conducted and newly prepared lymphocytes harvested from animals were analyzed by flow cytometry.

Supplementary Figure 2
**(a)** Sequence alignment results of the pET-32a-TgGAP45 plasmid. Compared with the nucleotide sequences of *T. gondii* GAP45 gene (Genbank: TGME49_223940), sequence analysis was carried out by the online Blast program (https://blast.ncbi.nlm.nih.gov/Blast.cgi). **(b)** Diagram of the construction of pET-32a-TgGAP45 plasmid.
