# Supplementary material for: PLGA nanoparticles as an efficient carrier in Toxoplasma GAP45: a more effective vaccine against acute toxoplasmosis than traditional ones
Source: Front Immunol. 2025 Jun 23;16:1600399. doi: 10.3389/fimmu.2025.1600399 (PMC12229859; doi:10.3389/fimmu.2025.1600399)
Supplement: Supplementary Figure 1 — Gating strategies for flow cytometry presented in the main article. Before cell sorting, adequate compensation was conducted and newly prepared lymphocytes harvested from animals were analyzed by flow cytometry. [file DataSheet1.docx]

# Supplementary Materials

| 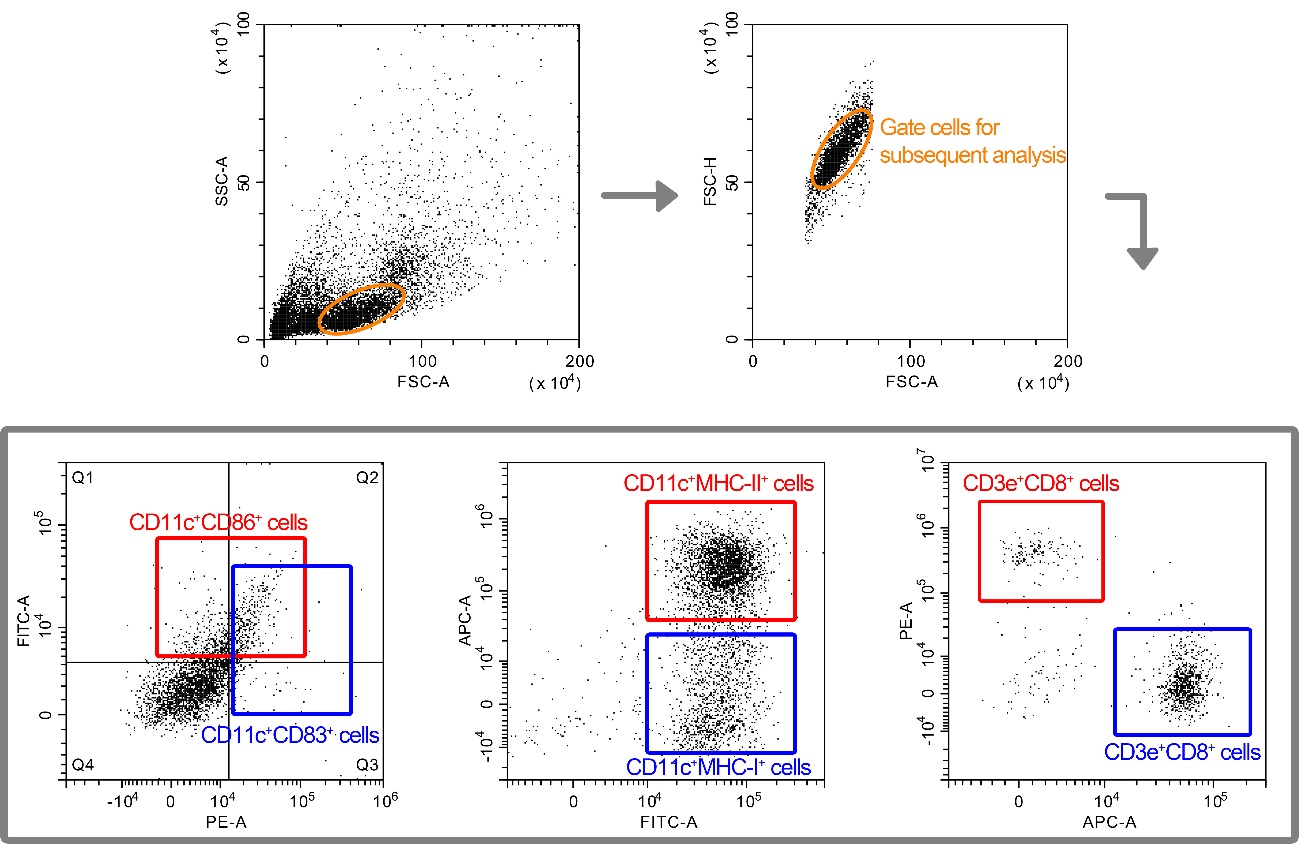 |
| --- |

**Figure S1.** Gating strategies for flow cytometry presented in main article. Before cell sorting, adequate compensation was conducted and newly prepared lymphocytes harvested from animals were analyzed by flow cytometry.

| 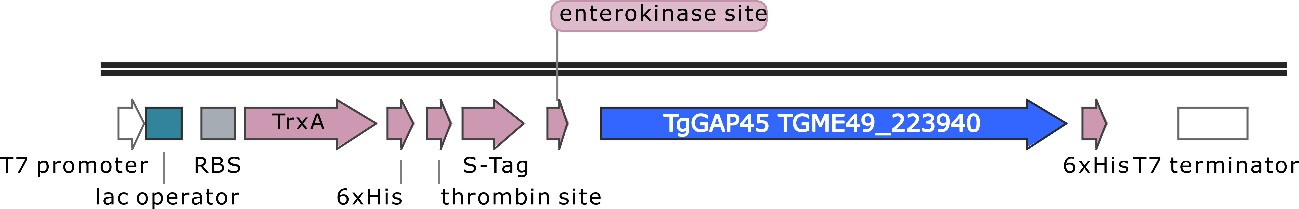 |
| --- |
| (a) |
| **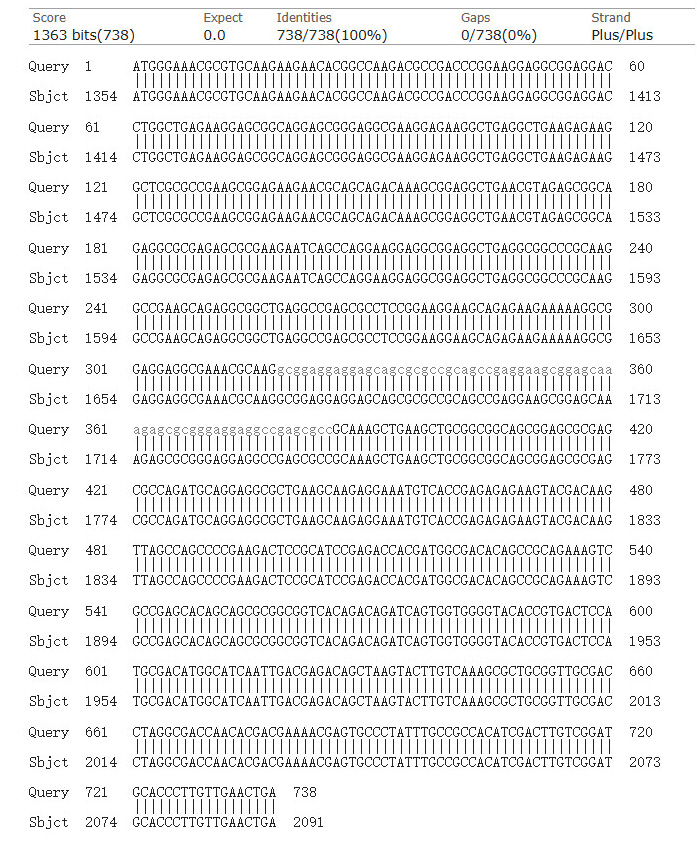** |
| (b) |

**Figure S2**. (a) Diagram of the construction of pET-32a-TgGAP45 plasmid. (b) Sequence alignment results of the pET-32a-TgGAP45 plasmid. Compared with the nucleotide sequences of *T. gondii* GAP45 gene (Genbank: TGME49_223940), sequence analysis was carried out by the online Blast program (https://blast.ncbi.nlm.nih.gov/Blast.cgi).
